# Implementation and feasibility of an interdisciplinary pediatric positive airway pressure adaptation program in a Brazilian public sleep clinic

**DOI:** 10.3389/frsle.2026.1791640

**Published:** 2026-04-16

**Authors:** Ila Linares, Jeane Xavier, Caroline Borginho, Clarissa Bueno, Anna Monazzi, Simone Fagondes, Renatha E. Rafihi-Ferreira, Melissa Xanthopoulos, Leticia Azevedo Soster

**Affiliations:** 1 Hospital das Clinicas da Faculdade de Medicina da Universidade de São Paulo, São Paulo, Brazil; 2 Universidade Federal do Rio Grande do Sul Faculdade de Medicina, Porto Alegre, Brazil; 3 Universidade de São Paulo Instituto de Psicologia, São Paulo, Brazil; 4 Children's Hospital of Philadelphia, Philadelphia, PA, United States

**Keywords:** adaptation, children, interdisciplinary, obstructive sleep apnea, positive airway pressure

## Abstract

**Introduction:**

The early diagnosis and treatment of pediatric obstructive sleep apnea (OSA) can improve academic performance of affected children, in addition to enhancing their cognitive and social development. Since the effective use of positive airway pressure (PAP) is limited by difficulties in adherence to the device, adaptation and engagement programs may improve outcomes in pediatric patients.

**Objective:**

The aim of this study is to describe an interdisciplinary program to improve PAP adherence among children with OSA in Brazil's public health system, also assessing its feasibility, acceptability, fidelity, and scalability based on implementation science frameworks, with the goal of informing sustainable clinical practice and public policy.

**Method:**

The program is based on the CPAP Program developed and implemented by the Children's Hospital of the Philadelphia Sleep Center. This prospective study will involve patients aged 1 year to 18 years treated at the children's sleep clinic who have a diagnosis of OSA and indication for PAP treatment. The main outcome measures will include (1) percentage of patients who return for a follow-up visit within 4 months of treatment initiation, (2) the median number of days from the initial visit to the first follow-up visit, (3) task analysis questionnaire, (4) objective data obtained from the device's memory card.

**Conclusion:**

The program proposed is expected to provide an integrated clinical service, optimizing the time of adaptation to PAP, increasing adherence rates, and reducing the costs associated with medical problems of untreated OSA.

## 1 Introduction

Pediatric obstructive sleep apnea (OSA) is defined as prolonged or intermittent complete or partial upper airway obstruction that disrupts normal ventilation or normal sleep patterns (American Academy of Sleep Medicine, 2023a). It affects 2–4% of children —particularly those aged 1 to 8 years (Kaditis, 2012) and those with neurodevelopmental conditions such as Trisomy 21, Prader-Willi syndrome, and Autism. Nocturnal manifestations include snoring, mouth breathing, restless sleep, enuresis, and increased nighttime awakenings, whereas daytime symptoms—such as irritability, inattention, fatigue, and motor agitation—may negatively affect neurocognitive development, academic performance, and psychosocial functioning (Ali et al., 1993; Guilleminault et al., 1981; Brooks and Topol, 2003; Chervin et al., 2002; Beebe, 2006).

Early diagnosis and treatment are essential to prevent these consequences and can improve academic performance of affected children, in addition to enhancing their cognitive and social development (Marcus et al., 2012). A range of treatment modalities are available, with adenotonsillectomy being the first-line treatment recommended by the AASM (Marcus et al., 2012; Mitchell et al., 2019). However, a significant percentage of children ranging from 13 to 29% will continue to have residual OSA after the surgical procedure; this percentage can reach 73% in the obese population (Tauman et al., 2006; Apostolidou et al., 2008) and is also much more prevalent in vulnerable individuals with neurodevelopmental conditions (Clements et al., 2021). Within this context, positive airway pressure (PAP), both continuous (CPAP) and bilevel (BiPAP), has been shown to be effective in the treatment of OSA (Parmar and Baker, 2019).

The effective use of PAP is limited by barriers in adherence (Uong et al., 2007; Koontz et al., 2003). Despite sample size variation, Perriol et al. (2019) reported that adherence can vary between 49% and 70% among the patients evaluated. In the study by Bhattacharjee et al. (2020), only 46.3% of the cohort met the adherence criteria (90 days of continuous use). It is therefore necessary to understand which variables can impair adaptation to PAP in the pediatric population. Demographic factors, including race and maternal education, appear to be significantly associated with PAP adherence in children (DiFeo et al., 2012). According to Bhattacharjee et al. (2020), children 4–6 years old and adolescent aged 15–18 years, may need more attention and support than other age groups. The suitability of the mask and the need for higher positive pressure also seem to influence treatment adherence.

Higher adherence rates are associated with early interdisciplinary support and close patient monitoring. This reflects a socioecological model in which medical, psychological, and environmental factors interact to shape treatment behaviors. Interdisciplinary teams—including sleep physicians, psychologists, respiratory therapists, and allied professionals—can address clinical, behavioral, and family-level determinants of PAP use, thereby strengthening treatment maintenance (Xanthopoulos et al., 2022).

The complex interaction between clinical, behavioral, and technical factors in pediatric OSA treatment, combined with the limited number of intervention studies targeting PAP adherence, underscores the need for structured implementation research. By integrating behavioral health principles with implementation science frameworks, this study goes beyond efficacy testing to examine feasibility, acceptability, fidelity, and contextual scalability in real-world practice. Although behavioral PAP interventions have been described internationally, no Brazilian studies have evaluated their implementation within the public health system. This study addresses this gap by adapting an established interdisciplinary model and assessing its acceptability and initial adherence outcomes in the Brazilian context.

In addition to assessing the primary clinical outcomes related to PAP adherence, this study will incorporate implementation indicators, as proposed by frameworks such as RE-AIM (Reach, Effectiveness, Adoption, Implementation, and Maintenance), a model designed to evaluate not only clinical effectiveness but also the real-world implementation, sustainability, and scalability of health interventions. Dimensions such as feasibility (e.g., the capacity to execute the protocol within the service), acceptability (e.g., caregivers' and professionals' perceptions of the program), fidelity (e.g., the degree to which the team adheres to the planned steps), and barriers to adoption or replication in other SUS (Sistema Unico de Saúde), the Brazilian public universal healthcare system, contexts will be explored. The inclusion of these indicators allows simultaneous assessment of clinical effectiveness and implementation outcomes, strengthening the program's scalability and policy relevance within publicly funded health systems.

## 2 Methods

### 2.1 Context and setting

As a reference in tertiary and multidisciplinary care from birth to adolescence, Instituto da Criança do Hospital das Clínicas da Faculdade de Medicina da Universidade de São Paulo (ICr-FMUSP) offers outpatient care, among other services. The childhood and adolescent sleep clinic of ICr-FMUSP is composed of doctors specializing in sleep, psychologists, and respiratory therapists.

### 2.2 Participants and eligibility criteria

Patients with an indication for PAP use who are between 1 and 18 years of age will be considered eligible to participate in the program for adaptation to PAP use.

PAP indication was defined according to established clinical standards. Patients were eligible when OSA was confirmed by overnight polysomnography (PSG) following AASM criteria (Marcus et al., 2012), particularly in cases of moderate-to-severe OSA or persistent OSA after adenotonsillectomy.

The presence of any neurological or clinical condition will not be a criterion for excluding patients from the program and will be properly addressed in statistical analysis. Parents need to have preserved cognitive capacity. The non-possession of a PAP device will be considered an exclusion criterion.

The parents of the participants will receive the free and informed consent form. The project (Ethical Clearance Certificate: 7.211.362) followed all local ethical guidelines and respects the Declaration of Helsinki and its amendments. The participants will not receive compensation.

### 2.3 Intervention phases

**Figure 1:**
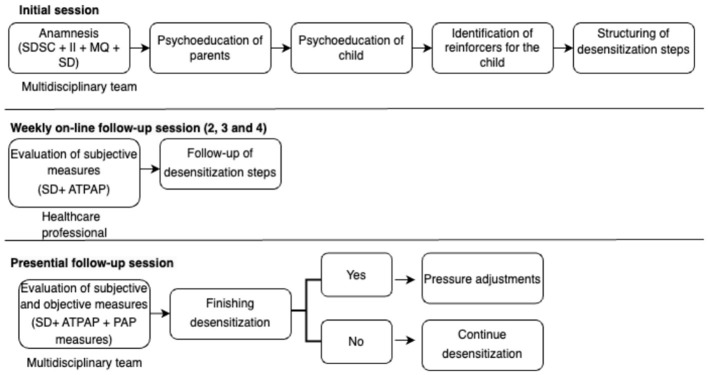
Flowchart of the interdisciplinary pediatric PAP adaptation program, illustrating the main stages from treatment indication to follow-up and adherence assessment.

Based on the CPAP Program of the Children's Hospital of the Philadelphia Sleep Center, this program will be implemented through a combination of strategies using an interdisciplinary approach. It should be noted that the program will start with the indication of PAP treatment and acquisition of a device by the patient's family. The overall structure and sequence of the pediatric PAP adaptation program are illustrated in Figure 1.

## 3 Visit structure and follow-up contacts

### 3.1 Initial visit

During the initial visit, the patients (caregivers and the child) will meet with the entire team, which will consist of a sleep physician, respiratory therapist, and psychologist. The aim of this consultation is to collect data about the child and family and to provide the parents with information about OSA, its treatment, and the program to which the child will participate.

Next, the child will receive psychoeducation using a booklet and video prepared (see supplementary material) for the pediatric population (direct demonstrations on the device, written information, and Supplementary videos prepared and produced by the team). Clarifying doubts, fears, and difficulties will be essential in all meetings of the team with the caregivers and children.

During this consultation, information about the child's preferred activities will be collected, which will be used in the adaptation program to promote distraction and motivation. Finally, the initial steps of the adaptation process will be agreed upon with the caregivers and the child (if possible).

### 3.2 Weekly monitoring

During monitoring of the adaptation process, all patients will have weekly follow-up video calls with the team (psychologist, respiratory therapist). These calls will not be recorded and they will address difficulties and value advances in PAP use. Families will receive an average of three video calls between the initial visit and the follow-up visit. Information obtained during weekly monitoring calls will be systematically recorded using structured monitoring forms. These records will include reports of device use, barriers to adherence, caregiver concerns, and any relevant clinical observations. The information will then be entered into the study database for monitoring adherence and intervention fidelity.

### 3.3 In-person follow-up visits

Follow-up visits will generally occur in person monthly, starting approximately 1 month after the initial visit, and will include the entire interdisciplinary team. The respiratory therapist will download the data card, and check the device (e.g., mask, filters, tubing, machine) to ensure that it is working correctly and with the prescribed settings.

The team will review the usage information with the family and provide individualized recommendations based on the objective data and family-reported experience. The team will assess reported side effects and benefits, answer any questions or concerns, and provide educational support.

## 4 Measures

During the initial interview, demographic and clinical data will be collected, including age, sex, primary and secondary diagnoses, and previous sleep treatment history. To comprehensively assess sleep patterns and behavioral aspects of PAP adherence, both objective and subjective measures will be used.

Objective measures will include PAP device data, such as average nightly use (hours/night), percentage of nights used, and leak indices, obtained through device downloads. Subjective measures will include standardized questionnaires and researcher-developed tools, as described below:

The Sleep Disturbance Scale for Children (SDSC; Bruni et al., 1996) is a 26-item parent-report questionnaire that evaluates six domains of pediatric sleep disorders: difficulties initiating and maintaining sleep, sleep-breathing disorders, arousal disorders, sleep–wake transition disturbances, excessive somnolence, and sleep hyperhidrosis. Items are rated on a 5-point Likert scale, and the instrument has been validated for Brazilian populations, showing good internal consistency (Cronbach's α >0.70) and clinical sensitivity (Ferreira et al., 2009).

The Motivational Questionnaire (MQ), developed by the research team, assesses parental and child motivation toward PAP treatment, exploring perceived benefits and barriers, readiness for change, and treatment confidence. It combines Likert-scale items with open-ended questions to capture expectations and experiences with medical devices.

The Initial Interview (II) is a semi-structured tool designed to obtain information about family routines, prior sleep habits, psychosocial context, and potential behavioral or sensory barriers to PAP use, including caregiver beliefs and emotional responses.

The Sleep Diary (SD), completed daily by caregivers, records bedtime, wake time, night awakenings, naps, PAP use duration, and contextual notes such as illness or routine changes, complementing objective adherence data. SD will be completed to ensure standardization, reliability, and continuity of sleep reporting across different age groups.

The Task Analysis for PAP Adherence (ATPAP; Koontz et al., 2003; Rafihi-Ferreira et al., 2020) operationalizes the child's behavior during PAP use into sequential, observable steps, allowing clinicians to identify barriers such as mask intolerance or anxiety and to tailor behavioral strategies.

Finally, the Satisfaction Inventory was developed by the research team to assess caregiver and acceptance and satisfaction with the program and its impact on daily routines and the quality of child–caregiver interaction.

Together, these instruments offer a multidimensional evaluation of adherence, motivation, and family engagement, integrating behavioral, emotional, and contextual factors relevant to the pediatric population treated in public health settings.

Interventions and evolution of the PAP program.

## 5 Desensitization

Based on the CPAP Program of the Children's Hospital of the Philadelphia Sleep Center, desensitization comprises gradual exposure, differential and arbitrary reinforcement, counterconditioning, and extinction. Gradual exposure will be performed by step-by-step presentation of the PAP device and its application according to the outlined sequence.

In the present protocol, structured daytime desensitization sessions are initiated with a minimum duration of 15–20 min. However, when clinically indicated—particularly in children presenting with sensory hypersensitivity, marked behavioral resistance, anxiety related to medical devices, or neurodevelopmental complexity—the duration of daytime exposure may be extended to 30–45 min, and, if necessary, up to 1 h.

Progression from daytime exposure to nighttime PAP use is not determined solely by fixed time criteria, but rather by the child's demonstrated behavioral tolerance, emotional regulation, and stability during exposure steps. Advancement occurs when the child exhibits sustained cooperation, reduced avoidance behaviors, and tolerance of the mask and airflow without significant distress.

Differential and arbitrary reinforcement consists of establishing contingent praise and positive events (differential reinforcement), including rewards of reinforcing stimuli (e.g., stickers). Counterconditioning consists of encouragement and praise for the child's participation in a favorite and distracting activity (e.g., TV, music, conversation, stories, film, computer game). These activities will be used to relax the child, so that the PAP device can then be presented.

Finally, interruption, blockade, or redirecting escape/avoidance behavior to extinguish these behaviors will also be part of this intervention. Table 1 describes how the desensitization process will take place.

**Table 1:** Description of desensitization steps.

Step	Desensitization step	Period	Duration
1	Exposure to the mask without headgear	Day	5 s, 10 s, 30 s, 1 min
2	Exposure to the mask with headgear	Day	1 min, 5 min,10 min, 15 min
3	Exposure to the mask with headgear and device turned on	Day	5 s, 10 s, 30 s, 1 min, 5 min
4	Exposure to the mask with headgear and device turned on	Night	1 min, 5 min, 10 min, 15 min
5	Exposure to the mask with headgear and device turned on	Night	More than 15 min

Despite standardization of the program, individualized care is essential to achieve the best outcomes. The identification of stressors and barriers in different settings (individual, family, and socio-environmental), as well as the perception of self-efficacy and motivation for implementing PAP, will result in a personalized intervention plan.

## 6 Outcomes

### 6.1 Main outcome

The primary outcome will be adherence to PAP treatment, assessed both objectively and through complementary process indicators. Objective adherence will be measured using data downloaded from the PAP device card or modem, including the average number of hours of use per night and the percentage of nights with at least 4 h of use, consistent with AASM recommendations (2023).

In addition, return to follow-up within 4 months of treatment initiation and the median number of days between the initial and first follow-up visit will be used as process indicators of adherence feasibility and treatment engagement.

### 6.2 Secondary outcomes

The secondary outcomes will encompass implementation, clinical, psychosocial, and process dimensions.

Implementation outcomes will follow the RE-AIM framework, assessing: (a) feasibility—proportion of participants completing all steps of the program; (b) acceptability—evaluated by caregiver and team satisfaction through the Satisfaction Inventory; (c) fidelity—assessed via a standardized checklist comparing team adherence to the protocol; and (d) sustainability—examined through the proportion of patients completing follow-up within the SUS and the time interval between follow-up visits.

Clinical outcomes will include changes in sleep quality, evaluated by the SDSC (Bruni et al., 1996), and objective PAP parameters, such as apnea–hypopnea index, mean pressure level, and oxygen desaturation metrics derived from baseline and follow-up assessments.

The ATPAP; Koontz et al., 2003; Rafihi-Ferreira et al., 2020) will also be applied to evaluate the progression of child behaviors necessary for successful PAP use. Psychosocial outcomes will include parental self-efficacy and motivation, as measured by changes in MQ scores between baseline and follow-up. Process outcomes will monitor treatment continuity through the number of teleconsultations or support contacts during the adaptation phase.

## 7 Fidelity monitoring and implementation evaluation

Adherence was categorized using thresholds commonly adopted in the PAP literature, particularly the criterion of ≥70% of nights with at least 4 h of use, which has been widely applied in adult populations and frequently adopted in pediatric studies (Koontz et al., 2003). Given the absence of standardized pediatric adherence classification levels, we operationalized adherence into three clinically meaningful categories to better capture gradations in treatment engagement: *excellent* (≥70% of nights and ≥4 h/night), *partial* (< 70% of nights or < 4 h/night), and *poor/non-adherent* (minimal or discontinued use).

If adherence did not meet the established criteria, the team will review the data, assess motivation and barriers, and develop a plan to improve adherence. If after 6 months of starting the adaptation program the child does not meet any of the 5 adaptation criteria, it will be considered an adaptation failure. If the child has met one or more of adaptation criteria, the program will be extended for 6 months. Finally, patients will be followed up at 3 months and then 6-month after reaching adherence criteria.

The implementation process will be assessed according to the RE-AIM framework (Reach, Effectiveness, Adoption, Implementation, and Maintenance; Glasgow et al., 1999; Silva et al., 2013). This approach allows the evaluation of both the clinical impact and the contextual feasibility of the intervention in a real-world setting.

In this study, RE-AIM—Reach will refer to the proportion of eligible families who accept to participate; Effectiveness will correspond to adherence and satisfaction outcomes; Adoption will capture the engagement of different professionals in the implementation of the protocol; Implementation will measure the fidelity of intervention delivery through standardized checklists; and Maintenance will be reflected in the continuity of PAP use and follow-up beyond the adaptation phase.

Fidelity of intervention delivery will be assessed using a structured protocol adherence checklist developed specifically for this program, based on established implementation fidelity frameworks (Carroll et al., 2007; Proctor et al., 2011). The checklist operationalizes the core components of the pediatric PAP adaptation protocol, including: (1) delivery of psychoeducation, (2) execution of graded desensitization steps, (3) caregiver training in behavioral strategies, (4) documentation of objective PAP data review, and (5) interdisciplinary coordination procedures (Table 2). Each component will be rated dichotomously (delivered/not delivered) and supplemented by qualitative notes regarding deviations or adaptations. Fidelity will be calculated as the proportion of planned components delivered per session and aggregated across cases.

**Table 2:** Study Timeline and Implementation Phases.

Phase	Timeframe	Implementation component	Key activities
Phase 1	Months 1–3	Preparation and capacity building	Training of interdisciplinary team; development of standardized materials; telehealth protocol structuring; alignment with SUS workflow
Phase 2	Months 4–18	Recruitment and baseline assessment	Enrollment of eligible participants; baseline clinical, motivational, and behavioral assessments; initiation of sleep diary; device acquisition
Phase 3	Up to 6 months per participant	PAP adaptation and behavioral intervention	Structured desensitization; parent-mediated exposure; weekly telehealth monitoring; monthly in-person follow-up; objective PAP data download and feedback;
Phase 4	3 and 6 months after adherence achievement	Post-adherence monitoring	Objective adherence reassessment; clinical follow-up; sustainability monitoring; caregiver satisfaction reassessment
Phase 5	Months 19–24	Data consolidation and evaluation	Data cleaning and verification; RE-AIM indicator analysis; fidelity assessment; statistical modeling of adherence trajectories

## 8 Statistical analyses

Data analysis will include descriptive and inferential statistics. Continuous variables will be summarized as means or medians, and categorical data as frequencies and percentages. The primary outcome, adherence to PAP (average nightly hours and percentage of nights with ≥4 h of use from device downloads), will be analyzed using paired *t*-tests or Wilcoxon tests to compare baseline and follow-up, and linear mixed models to evaluate adherence trajectories over time.

For secondary outcomes, changes in SDSC and MQ scores will be compared using paired tests, and associations between motivation, self-efficacy, and adherence will be explored with correlation and regression analyses. Implementation indicators from the RE-AIM framework will be reported descriptively.

Missing data under 5% will be handled by complete-case analysis; otherwise, multiple imputation will be applied. Analyses will be two-tailed with α = 0.05 and performed in R or Stata.

Given the clinic's recruitment capacity, this implementation-focused cohort will enroll up to *n* = 20 participants. Although the primary aim is to evaluate feasibility and implementation outcomes, an exploratory power analysis was conducted for the primary clinical endpoint (proportion achieving ≥70% nights of PAP use). Assuming a two-sided α = 0.05 and 80% power, a sample of 20 participants allows detection of a minimum detectable difference (MDD) of approximately 0.35–0.40 in adherence proportion (e.g., from 50% under usual care to approximately 85–90%) (Kang et al., 2019).

## 9 Implementation, challenges and feasibility outcomes

(Xanthopoulos et al. 2022) described interventions to address the challenges identified after implementing the PAP adaptation program. Since the present study is based on the program used by the Children's Hospital of the Philadelphia Sleep Center, the possible measures to be established in light of the aspects raised by the authors are described.

Feasibility was evaluated according to pre-defined criteria adapted from Bowen et al. (2009), encompassing both patient- and therapist-related indicators. Patient-related feasibility measures included: (a) number of eligible participants sufficient to initiate at least one cohort per semester (≥8 families); (b) proportion attending the first session (target ≥70%); (c) mean number of follow-up contacts per patient (≥3); and (d) attrition rate below 25% (Table 3).

**Table 3:** Structuring possible challenges and potential solutions (table based on the article by Xanthopoulos et al., (2022).

Points of attention	Risk of occurrence	Suggested solutions
Screening for eligible patients	low	Dissemination among the outpatient clinics of ICr
Coordination of the support team after the initiation of PAP use	low	Following the program with the responsibilities of each professional standardized recording of the content of each visit
Consistent follow-up	medium	Standardization of intervals between visits standardization of the topics to be covered in each visit
Communication between team members	low	Weekly meetings with the interdisciplinary team

Therapist-related feasibility measures included perceived credibility, workload compatibility, and willingness to continue using the protocol after study completion, assessed through semi-structured interviews. These indicators were selected to capture both the internal feasibility of the intervention and its external applicability to routine clinical practice within Brazil's public health system (SUS).

## 10 Discussion

There are no structured and validated protocols in Brazil for interdisciplinary adaptation to PAP in children with OSA — although this remains one of the major challenges reported in both national and international literature. So, this study describes an interdisciplinary program for adaptation to PAP use that will be implemented in a pediatric population at ICr-FMUSP.

The program aligns with international guidelines (American Academy of Sleep Medicine, 2023b) and addresses a critical need within the SUS. Regarding its structure, it is organized in steps so that caregivers and the child can gradually achieve the proposed objectives. Its goal is to provide an integrated clinical service, optimizing the time required for PAP adaptation, increasing adherence rates, and reducing costs associated with medical problems caused by untreated OSA. The training program seeks to promote PAP adherence by distracting from uncomfortable sensations, gradually exposing the child to the steps necessary for use of the device and inducing a relaxed positive state by offering preferred activities, counterbalancing emotional arousal. Introducing PAP can also be stressful for parents who may also attempt to avoid using the device due to the often negative interaction with their child. Further, there is also a learning curve for parents in using and feeling comfortable with the device itself. Introducing PAP with repeated, playful daytime exposures can also make the implementation less aversive and positive for caregivers, as well as gives them practice at improving their skills in using the device. It can also improve their self-efficacy. Caregiver self-efficacy is an important component of implementing PAP (Xanthopoulos et al., 2017).

Caregivers who seek to implement PAP use often react to the child's discomfort by trying to calm him/her and by using verbal explanations. This approach can reinforce the child's discomfort, as well as escape and avoidance behaviors (Koontz et al., 2003). With the repetition of this interaction pattern, the child develops a varied and persistent repertoire of escape behaviors from the mask and caregivers will thus give up implementing PAP use (Slifer, 2011).

As part of the interdisciplinary support, psychology, specifically the behavioral approach, contributes to PAP adherence since it is aimed at reducing or eliminating escape/avoidance behaviors of the discomforting feeling and at positively reinforcing the use of the device, as well as sustained adherence to the mask and to the air pressure (Koontz et al., 2003). As the child experiences a reduction in anxiety and exhibits fewer avoidance behaviors, compliance behaviors may be strengthened. Favorite items or events are provided in response to the child's efforts to cooperate with and tolerate the mask and air pressure. Positive parenting strategies such as praise and social reinforcement will also be utilized (Koontz et al., 2003).

The use of a preferred activity during exposure creates conditions for greater willingness to comply with the training. The child's age seems to have a special influence when sleep patterns are evaluated, which can be considered typical or atypical for the respective age group (Montgomery-Downs et al., 2004). Thus, both the diagnosis and the treatment will also vary according to age group, i.e., the language and strategies of the program will require adjustments and adaptations to the needs of each case.

Adherence to pressure therapy is extremely variable. In the adult population, adherence to PAP is defined as using the pressure device for more than 4 h per night on 70% of nights over a period of 30 consecutive days (Naik et al., 2019). However, there is no consensus regarding the correct period of adherence to PAP treatment in the pediatric population. A recent review showed that adherence in the pediatric population is < 60% among patients, with a mean time of use between 4.0 and 5.2 h per night (Watach et al., 2020). This concept of 4 h of use is highly questionable in the pediatric population (Hady, 2021) since the child needs a longer period of sleep. The approach of PAP device used on more than 70% of nights and for more than 6 h per night covers the specificity of children's and adolescent's sleep.

Expected limitations within the Brazilian context include structural and contextual barriers that may influence the implementation and scalability of the program. These challenges encompass low income profile, limited access to technology, cultural barriers affecting interaction with medical devices, and the shortage of interdisciplinary teams in SUS services outside tertiary care centers. SUS is a nationwide public system that provides universal and free access to care but is characterized by marked heterogeneity in infrastructure, availability of specialized services, and professional training across regions. Such factors may constrain the feasibility and generalizability of adherence protocols, underscoring the importance of tailoring implementation strategies to local resources, training capacities, and sociocultural characteristics to ensure sustainable outcomes.

To address these contextual limitations, the program incorporates several mitigation strategies aimed at enhancing feasibility and scalability within resource-constrained public health settings. These include telehealth follow-up to reduce transportation burden and improve access for families living far from tertiary centers, as well as flexible scheduling to accommodate caregivers' work routines and minimize absenteeism. Parent-mediated exposure strategies are implemented to empower caregivers to conduct desensitization procedures at home, thereby decreasing reliance on frequent in-person visits. The program also utilizes low-cost behavioral materials—such as printed visual aids, reinforcement charts, and structured routines—to ensure affordability and replicability. In addition, integration with the existing multidisciplinary clinic flow optimizes coordination among professionals and minimizes additional system burden. Standardized documentation protocols are used to improve continuity of care in high-demand services, and weekly interdisciplinary meetings are conducted to enhance communication and prevent fragmentation of care.

Consistent with Bowen et al. (2009), feasibility in this study refers not only to the technical implementation of the intervention, but also to its adaptability, acceptability, and sustainability within a real-world clinical environment. The inclusion of standardized feasibility criteria and RE-AIM indicators provides an empirical framework to assess whether the program can be realistically maintained and scaled in other public pediatric services. Conducting the study within the SUS—a system marked by resource limitations, heterogeneous patient populations, and high service demand—adds ecological validity and tests the model's resilience under non-ideal conditions.

Importantly, therapist feedback and high family engagement will inform subsequent refinements of the protocol, including potential task-shifting strategies (e.g., telehealth follow-ups or paraprofessional training) to enhance scalability. Future studies should evaluate long-term sustainability and cost-effectiveness to inform national policy and training frameworks in pediatric sleep health.

According to some authors, the medical costs of treating children with OSA are 215% higher in the year prior to diagnosis when compared to their healthy peers; these higher costs are due to hospitalizations, emergency room visits, and high rates of respiratory tract infections (Jennum et al., 2013). Although there are no studies that quantify the overall cost of untreated or undertreated pediatric OSA, the estimated annual costs of treating the consequences of adult OSA are billionaire (Tarasiuk and Reuveni, 2013).

In Brazil, no studies have quantified the economic burden of untreated pediatric OSA. However, international evidence demonstrates that children with OSA have significantly higher healthcare utilization prior to diagnosis, including increased hospitalizations and emergency visits (Jennum et al., 2013). Given the clinical, behavioral, emotional, and academic burdens associated with pediatric OSA, further studies involving representative samples are essential to evaluate the effectiveness of PAP adherence programs and strengthen the generalizability of findings. The inclusion of a multidisciplinary team—encompassing psychology, pediatrics, and other allied health fields—enhances the external validity of this protocol and positions it as a potential model for other services. Highlighting similar initiatives for PAP adaptation in children outside the United States can also situate this program as part of a broader global effort to promote equitable and evidence-based pediatric sleep care.
