# Supplementary material for: Implementation and feasibility of an interdisciplinary pediatric positive airway pressure adaptation program in a Brazilian public sleep clinic
Source: Front Sleep. 2026 Apr 16;5:1791640. doi: 10.3389/frsle.2026.1791640 (PMC13130487; doi:10.3389/frsle.2026.1791640)
Supplement: Supplementary file 1 [file Supplementary_file_1.docx]

**Supplementary material**


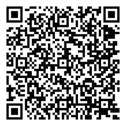


1. Psychoeducation - booklet
2. Psychoeducation - video

https://www.youtube.com/watch?v=4CU13eiP_BU
